# Birth and Death Rate in Large Cities

**Published:** 1881-08

**Authors:** 


BIRTH AND DEATH RATE IN LARGE CITIES.—The old adage of, God made the country, and man the town, is scarcely anywhere so strikingly exemplified as in the comparative increase of population in the country or rural districts over the larger communities and commercial centers like London, New York and some other cosmopolitan cities.

The death rate is so greatly in excess of the births in these large and densely populated communities, that they would retrograde rapidly in population but for the streams of immigration so constantly flowing into them from the rural districts, contiguous to them, and from foreign lands. While the population of the whole earth is constantly increasing at a slow but steady rate, that of the large cities is decreasing, *i. e.* from natural causes or from death. The well-known greater fatality of city life to children from summer complaints and contagious and infectious diseases, would ultimately operate against their increase or growth in population, even if the proportion of births to the deaths were the same as in country districts! But with the birth and death rates of those large centers before us, it is easy to perceive that, without immigration, those large communities would certainly in process of time die out or become extinct.

An important subject for the sanitarian and philanthropist here opens up, and though the field is an inviting one, we are compelled to forego its exploration for the present. On an early occasion however, we hope to lay the published statements of a distinguished gentleman before our readers, as to the radical causes operating upon the birth rate in city and in some small communities, which if they should become wide spread would contribute largely towards the extinction of some of the races, as they are even now sapping the life blood of religion and sound morablity.

